# Comprehensive behavioral characterization of an APP/PS-1 double knock-in mouse model of Alzheimer's disease

**DOI:** 10.1186/alzrt182

**Published:** 2013-05-24

**Authors:** Scott J Webster, Adam D Bachstetter, Linda J Van Eldik

**Affiliations:** 1 Sanders-Brown Center on Aging, 800 S. Limestone, University of Kentucky, Lexington, KY 40536, USA; 2 Department of Anatomy and Neurobiology, 800 S. Limestone, University of Kentucky, Lexington, KY 40536, USA

**Keywords:** Alzheimer's disease, amyloid precursor protein/presenilin-1, motor behavior, anxiety behavior, cognition, learning and memory, spatial reference memory, recognition memory, transgenic mouse model

## Abstract

**Introduction:**

Despite the extensive mechanistic and pathological characterization of the amyloid precursor protein (APP)/presenilin-1 (PS-1) knock-in mouse model of Alzheimer's disease (AD), very little is known about the AD-relevant behavioral deficits in this model. Characterization of the baseline behavioral performance in a variety of functional tasks and identification of the temporal onset of behavioral impairments are important to provide a foundation for future preclinical testing of AD therapeutics. Here we perform a comprehensive behavioral characterization of this model, discuss how the observed behavior correlates with the mechanistic and pathological observations of others, and compare this model with other commonly used AD mouse models.

**Methods:**

Four different groups of mice ranging across the lifespan of this model (test groups: 7, 11, 15, and 24 months old) were run in a behavioral test battery consisting of tasks to assess motor function (grip strength, rotor rod, beam walk, open field ambulatory movement), anxiety-related behavior (open field time spent in peripheral zone vs. center zone, elevated plus maze), and cognitive function (novel object recognition, radial arm water maze).

**Results:**

There were no differences in motor function or anxiety-related behavior between APP/PS-1 knock-in mice and wild-type counterpart mice for any age group. Cognitive deficits in both recognition memory (novel object recognition) and spatial reference memory (radial arm water maze) became apparent for the knock-in animals as the disease progressed.

**Conclusion:**

This is the first reported comprehensive behavioral analysis of the APP/PS1 knock-in mouse model of AD. The lack of motor/coordination deficits or abnormal anxiety levels, coupled with the age/disease-related cognitive decline and high physiological relevance of this model, make it well suited for utilization in preclinical testing of AD-relevant therapeutics.

## Introduction

A large number of mouse models have been genetically engineered in attempts to model different aspects of the etiology and pathology of Alzheimer's disease (AD). While all transgenic mouse models generated to date fail to replicate completely the pathology observed in human AD, they have offered valuable insight into the molecular mechanisms of AD and have provided a useful preclinical platform with which to test potential AD therapeutics [1]. Many of the currently used AD mouse models are generated by random exogenous insertion of genetic material into the host genome to produce overexpression of a particular protein of interest. While this approach is relatively straightforward and provides a convenient way to examine a gene/protein of interest, ectopic overexpression of a gene can lead to off-target complications unrelated to the disease process that can complicate experiments aimed at evaluating novel AD therapeutics. For example, ectopic overexpression of even a wild-type (WT) transgene can evoke cellular, anatomical, and behavioral abnormalities [2-7]. One way to bypass these off-target complications common to transgenic mice is to knock-in (KI) the gene of interest into a specific genetic locus in the mouse genome.

The APP^NLh/NLh^ × PS1^P264L/P264L ^double gene-targeted knock-in (APP/PS1 KI) mouse takes advantage of this KI gene-targeted insertion with selective point mutations in amyloid precursor protein (APP) and presenilin-1 (PS-1) genes linked to familial AD pathology [3,8-11]. In general, the use of the genetic KI strategy potentially increases the fidelity of a model system of a relevant disease process. A case in point is the APP/PS1 KI mouse, which replicates much of the amyloid-dependent pathologies seen clinically in AD. For example, nearly identical profiles of amyloid processing exist between observed AD patients and these APP/PS1 KI mice [12]. Further, these mice exhibit progressive amyloid deposition starting at 6 months of age that increases linearly over time, so that by 18 months of age they show many dense amyloid deposits in regions such as the hippocampus and dentate gyrus [11-14]. These amyloid depositions consist of both neuritic and non-neuritic plaques with high similarities to those seen in human AD [12]. Other observed pathological changes for this model that are relevant to the pathogenesis of AD include: increased oxidative stress and metabolic disturbances starting as early as 1 to 2 months of age, reduction of neuronal L-type calcium channel activity in 14-month-old mice, impaired hippocampal LTP, and age-related increases in reactive gliosis and proinflammatory cytokine production [11-13,15-17].

Despite the extensive biochemical and mechanistic characterization of this APP/PS1 KI mouse model, less is known about AD-relevant behavioral/cognitive deficits of this model. One report describes cognitive deficits in 14-month-old APP/PS1 KI mice in a one-way active avoidance task [16]. Another study showed an age-dependent cognitive impairment in a Stone T-maze task [18]. However, to date a comprehensive behavioral analysis of this AD model has not been reported. Little is therefore known about the baseline behavioral profile and the temporal onset of behavioral impairments in this APP/PS1 KI model in a series of standard behavioral tasks. Here we seek to fill this void in knowledge by examining the cognition/behavioral profile (motor behavior, anxiety-related behavior, and cognitive function) across the lifespan of the APP/PS1 KI mouse using a cross-sectional design (age groups of 7, 11, 15, and 24 months old). We report here that the APP/PS1 KI mouse shows no motor deficits or abnormal anxiety levels at any of the ages tested. In addition, the APP/PS1 KI mouse shows an age-dependent development of cognitive deficits in two different memory domains relevant to AD: spatial reference memory and recognition memory [19-21].

## Methods

### Mice

Four different age groups (7, 11, 15, and 24 months) of APP^NLh/NLh^ × PS1^P264L/P264L ^mice were used in this study. This mouse model was originally developed at Cephalon [11] using gene-targeted KI technology to introduce the Swedish FAD K670N/M671L point mutations, humanize the mouse β-amyloid sequence (NLh), and introduce a proline to leucine (P264L) mutation in the mouse PS-1 gene [8,9]. A useful characteristic of this model is that because an endogenous promoter drives the expression of each gene, development of AD-like pathology occurs in the absence of APP or PS1 overexpression. Mice were maintained on a CD-1/129 background, and WT mice were obtained from heterozygous APP-PS1 mating pairs and maintained as a separate line for more than 20 generations of inbreeding, for use as controls. All mice were genotyped by PCR analysis of tail-snip DNA to monitor for the maintenance of the appropriate genotype [22].

Animal protocols followed the principles and practices outlined in the Guide for the Care and Use of Laboratory Animals, and were approved by the Institutional Animal Care and Use Committee of the University of Kentucky. All behavioral testing was performed in the University of Kentucky Rodent Behavior Core. There were no observable differences in mortality rates between genotypes in this study, and behavioral data for both genders were combined after observing no differences between the behavioral responses of male and female mice for any age group tested.

### Grip strength

Forelimb grip strength was measured in grams of resistance using a digital force-gauging apparatus (Animal Grip Strength System; San Diego Instruments, San Diego, CA, USA). Forelimb strength was measured by holding the mouse by the nape of the neck and by the base of the tail. The forelimbs were placed on the tension bar pad, and the mouse was pulled back gently until it could no longer grip the bar and was forced to release its hold on the grip pad. The resistance was automatically calculated in real time with the digital force-gauging apparatus and the maximal resistance achieved by each mouse was reported as the final grip strength.

### Rotor rod

The mice were placed on a rotating rod (3.18 cm diameter) in lanes 11.5 cm wide to maintain the animal in the same direction while the bar is rotating (ROTOR-ROD™ System; San Diego Instruments). The bar is 46 cm from the floor of the apparatus and the bar's speed of rotation was gradually and linearly increased from 0 to 40 rpm across the 5-minute trial. Both the latencies (seconds) and the distance (cm) at which the mice were able to maintain their balance on the bar were then recorded automatically using beam break technology.

### Beam walk

The beam walk protocol used in this study has previously been described [23]. Briefly, mice were trained to walk along an 80 cm long and 3 cm wide beam elevated 30 cm above the bench by metal supports to reach an enclosed goal box. Mice were placed on the beam at one end and allowed to traverse the beam to reach the goal box. This was repeated using decreasing size (3 cm, 2 cm, and 1 cm) beams. Foot slips were scored when one or both hind limbs slipped from the beam.

### Open field activity

Mice were placed in a multi-unit open field maze (San Diego Instruments) with field chamber (50 cm long × 50 cm wide), and activity was recorded using EthoVision XT 8.0 video tracking software (Noldus Information Technology, Leesburg, VA, USA). Each 50 cm × 50 cm unit was digitally divided into 25 quadrants of equal size (nine central and 16 peripheral) using EthoVision XT 8.0 video tracking software. The nine central quadrants are collectively referred to as the center zone and the 16 peripheral quadrants are collectively referred to as the peripheral zone as previously described [24]. Data were collected continually for 30 minutes and the distance traveled (cm), velocity (cm/second), and time spent in the center zone versus the peripheral zone were all recorded and scored automatically. The open field task is a popular model for assessing ambulatory movement and anxiety-like behaviors in response to a novel environment. Distance traveled and movement speeds are measures of ambulatory movement, whereas the amount of time spent in the center zone versus the peripheral zone is a measure of anxiety levels due to the rodent's natural thigmotaxis behavior when frightened [25].

### Elevated plus maze

An elevated plus maze (San Diego Instruments) was used to assess anxiety-related behavior in response to a potentially dangerous environment. The elevated plus maze consists of four arms (two enclosed arms and two open arms) elevated 100 cm above the floor. Anxiety-related behavior was defined as the degree to which the subject avoided the open arms (perceived unsafe arms) of the maze, preferring the closed arms (perceived safe arm) of the maze. Each mouse was placed in the center of the maze and the amount of time spent in each arm was recorded automatically by EthoVision XT 8.0 video tracking software (Noldus Information Technology).

### Radial arm water maze

The radial arm water maze (RAWM) task can be used to measure both spatial working memory [26,27] and spatial reference memory. The maze procedure used in this study was designed as a rapid measure of spatial reference memory and has been previously described in detail [28]. Briefly, the maze consisted of six arms 160 cm in diameter with arm length 30 cm and common circular swim area of 40 cm. The pool was filled with water until the level was approximately 2 cm above (covering) a clear (invisible) 10-cm circular platform. The platform was placed in the back of an arm approximately 7 cm away from the side and back walls. The pool was located in the center of a room and enclosed by a black curtain. Geometric extra-maze visual cues were fixed throughout the study on three sides of the curtains.

The RAWM protocol consisted of a 2-day testing paradigm. A staggered training schedule was used, running the mice in cohorts of five or six mice, while alternating the different cohorts through the trials over day 1 and day 2 of the test. This alternating protocol was used to avoid the learning limitations imposed by massed subsequent trials and to avoid fatigue that may result from consecutive trials. During block 1 (six trials) and block 2 (six trials), mice were trained to identify the platform location by alternating between a visible and a hidden platform in the goal arm, with three hidden platform trials and three visible platforms. Block 3 consisted of three trials all with a hidden platform. For day 2, mice were tested in three blocks of five trials each (15 total trials), with only the hidden escape platform employed, forcing the mice to use a spatial strategy to identify the goal arm location.

Data are presented as the average errors per block, similar to the methods described in [28], and only errors during the hidden platform trials are included in the analysis, as they represent the spatial memory component of the RAWM task. Errors were scored each time the subject entered the arm not containing the platform. An arm entry was defined as the mouse's whole body moving past the threshold of the entrance to an arm. After reaching the platform, the mouse was allowed to remain on it for 10 seconds and was then removed, dried, and placed in a warming cage until the initiation of that mouse's next trial. For each subsequent trial the mouse was released from a different start arm into the maze and allowed to locate the platform. Platform location remained constant throughout testing. RAWM performance was recorded and scored using EthoVision XT 8.0 video tracking software (Noldus Information Technology). Every arm entry for each animal was recorded and reviewed to ensure that the mice did not employ nonspatial/kinesthetic strategies (that is, chaining) to solve the task. No chaining response (defined as a mouse consecutively entering three (or more) adjacent arms to solve the task) was observed for either genotype.

### Novel object recognition

This task of recognition memory utilizes the fact that animals will spend more time exploring a novel object compared with an object that they are familiar with in order to satisfy their innate curiosity/exploratory instinct. The test apparatus consisted of an open field box measuring 39.4 cm × 78.8 cm change confirmed. in diameter, and all sessions were video-recorded. On day 1 the animal was allowed to explore the open field box for a 15-minute time period. The following day the animals were each exposed to a 10-minute information session (that is, the A/A session with identical objects present). This information session was followed by a 1-hour delay during which the animals were returned to their home cages. After the delay the animals performed a 10-minute dissimilar stimuli session (A/B). The objects were made of hard plastic and had previously been counterbalanced to control for any object preference bias. The total amount of time spent with each object was recorded and scored using fully automated EthoVision XT 8.0 video tracking software (Noldus Information Technology).

The time spent was operationally defined as occurring when an animal directed its nose to the object at a distance <2.0 cm and/or by the animal touching the object with its nose or mouth. Data are presented as the *D*^2 ^discrimination index. The *D*^2 ^index is a common measure of discrimination between novel and familiar objects, and is considered one of the most reliable measures of discrimination because it corrects for total exploratory activity of each animal [29]. The *D*^2 ^index is calculated for an A/B session by examining the difference in time spent exploring the novel and familiar objects divided by the total exploration time for both objects:

$$D^{2}=\left(\text{novel }-\text{ familiar}\right)/\left(\text{novel }+\text{ familiar}\right)$$

The *D*^2 ^index thus corrects for total exploratory behavior of each mouse.

### Statistical analysis

All statistical analyses were performed using GraphPad Prism Version 5.00 (GraphPad Software, San Diego, CA¸USA). In the cross-sectional study design, experimental groups consisted of two genotypes at four different ages: 7 months, 11 months, 15 months, and 24 months. Each group used a ~50:50 ratio of males to females. The number of mice used per group was 5 to 12 mice per group. Unless otherwise indicated, for WT mice there were 10 aged 7 months, nine aged 11 months, five aged 15 months, and 10 aged 24 months; and for KI mice, there were nine aged 7 months, five aged 11 months, 10 aged 15 months, and five aged 24 months.

Comparison between individual experimental groups was performed by unpaired *t *test where appropriate. Regression analysis for RAWM and novel object recognition **(**NOR) cognitive data was modeled via linear regression in GraphPad with genotype, age, and errors/*D*^2 ^index as independent variables and using a 95% confidence interval. Data are expressed as the mean ± the standard error of the mean. Differences between means from experimental groups were considered significant at the *P *<0.05 level.

## Results

### Vestibulomotor behavior

**Figure 1:**
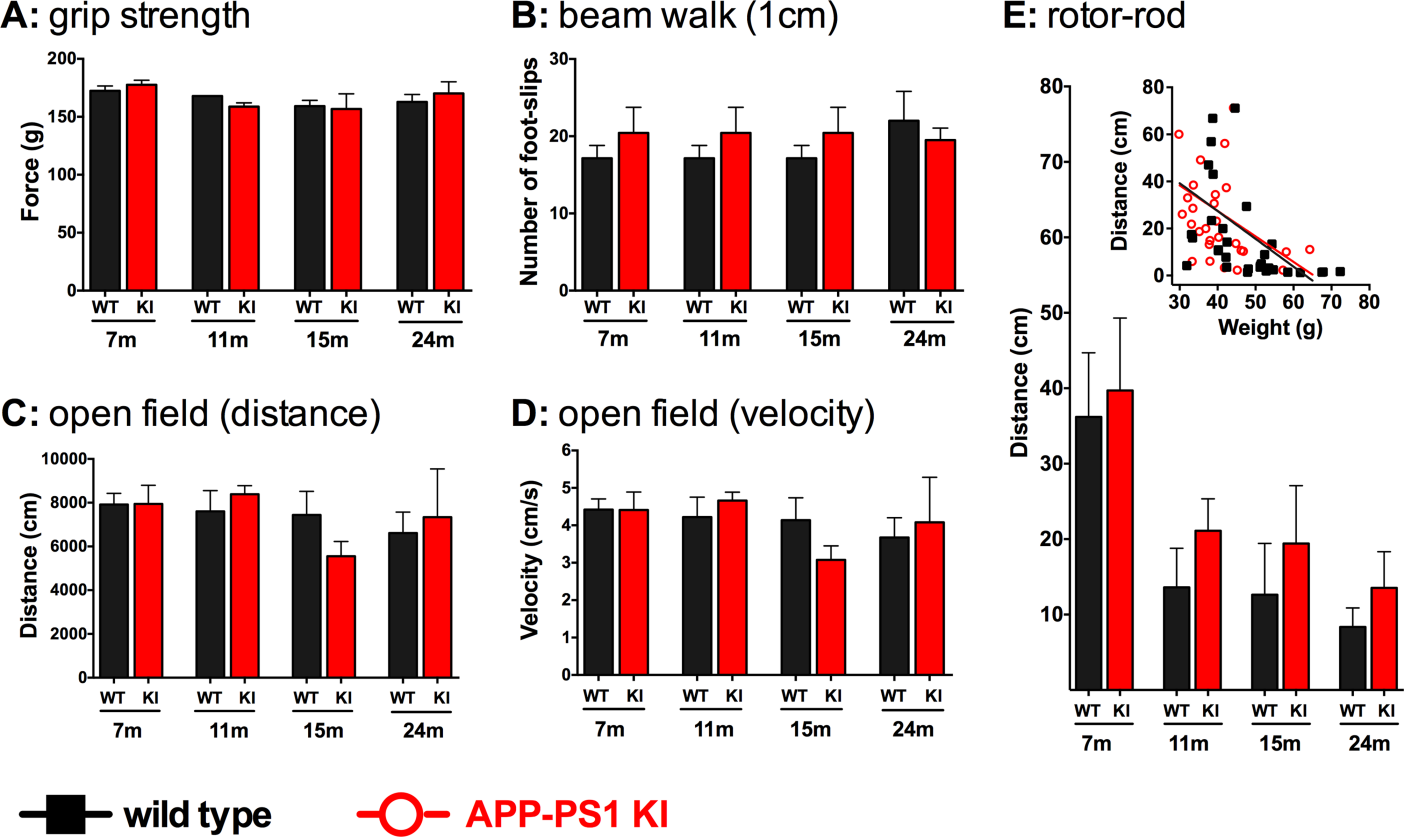
**Motor behavior of APP/PS1 KI and wild-type control mice**. **(A) **No differences in grip strength are observed between amyloid precursor protein/presenilin-1 knock-in (APP/PS1 KI) mice and their wild-type (WT) counterparts in any age group. **(B) **No performance differences are observed between APP/PS1 KI and WT mice for any age group in the beam walk task. **(C), (D) **Open field performance shows no significant differences in ambulatory movement, as assessed by (C) distance traveled or(D) movement velocity, between APP/PS1 KI and WT mice for any age group. **(E) **Rotor rod performance shows no differences between APP/PS1 KI and WT mice for any age group. However, performance is negatively correlated with weight (E insert) for the WT animals (*P *= 0.004) and is almost significantly correlated for APP/PS1 KI mice performance (*P *= 0.052). Number of mice: 10 WT and nine KI mice aged 7 months; five WT and eight KI mice aged 11 months; eight WT and eight KI mice aged 15 months; and eight WT and five KI mice aged 24 months.

The vestibulomotor behavior of APP/PS1 KI and WT control mice is presented in Figure 1. As shown in Figure 1A, no differences were observed between APP/PS1 KI and WT mice in grip strength performance. Further, the grip strength remained consistent across the age groups for both APP/PS1 KI mice and their WT counterparts.

No differences in beam walk performance were observed between genotypes or for any age group of mouse tested (Figure 1B). The number of foot slips for both APP/PS1 KI and WT mice increased as the size of the bar decreased. APP/PS1 KI mice made 1.92 ± 0.26 foot slips compared with 1.46 ± 0.37 foot slips for the WT mice crossing the 3 cm beam. For the 2 cm beam, the APP/PS1 KI mice made 9.69 ± 1.09 foot slips while the WT mice made 8.36 ± 0.70 foot slips. Figure 1B also shows the APP/PS1 KI and WT mice performance on the 1 cm beam (APP/PS1 KI mice had 20.28 ± 1.54 foot slips compared with 17.92 ± 1.00 foot slips for WT mice).

The open field behavioral task to assess ambulatory movement showed no difference between APP/PS1 KI and WT mice in distance traveled (Figure 1C) or in movement speed (Figure 1D) for any age group. Similarly, no differences in habituation behavior were observed in the open field task for either genotype or for any age group (data not shown).

Finally, no differences were observed at any age group between APP/PS1 KI and WT mice in rotor rod performance (Figure 1E), although the younger animals (7 months old) performed this task somewhat better than the older age groups. Interestingly, performance was negatively correlated with the weight of the animals (Figure 1E, insert). Specifically, WT mice performance showed a highly significant negative correlation with weight (*P *= 0.004), and APP/PS1 KI mice showed a similar pattern that did not reach statistical significance (*P *= 0.052).

### Anxiety-related behavior

**Figure 2:**
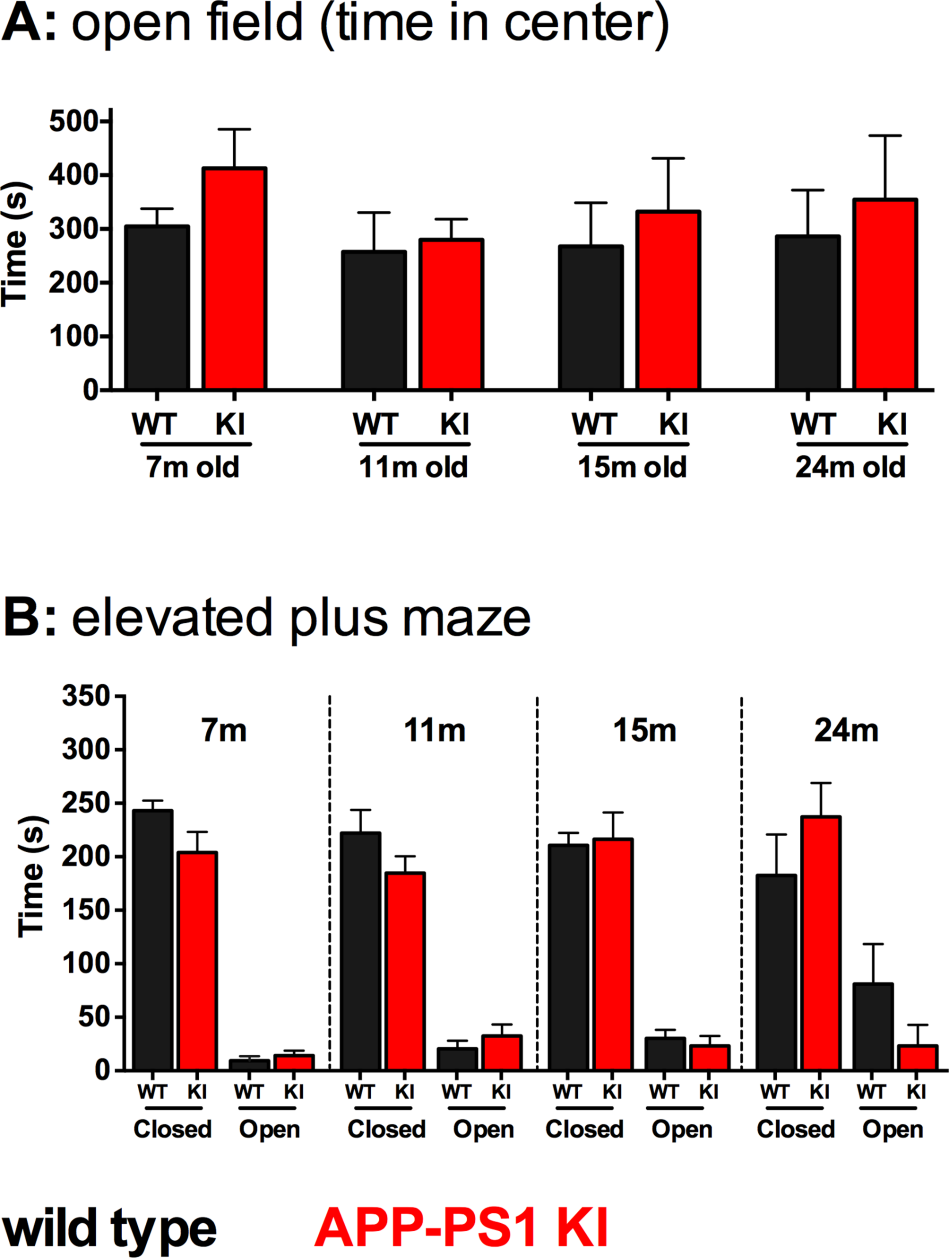
**Anxiety-related behavior of APP/PS1 KI and wild-type control mice**. **(A) **No difference in the time spent exploring in the center zone between the amyloid precursor protein/presenilin-1 knock-in (APP/PS1 KI) mice and the wild-type (WT) mice was observed in any age group. **(B) **Elevated plus maze anxiety behavior is also similar between APP/PS1 KI and WT mice for all ages. Number of mice: 10 WT and nine KI mice aged 7 months; five WT and eight KI mice aged 11 months; eight WT and eight KI mice aged 15 months; and eight WT and five KI mice aged 24 months.

The anxiety-related behavior of APP/PS1 KI and WT mice was assessed by two behavioral tests: analysis of time spent in the center zone versus peripheral zone of the open field arena; and the elevated plus maze task. As shown in Figure 2A, no differences were seen between APP/PS1 KI and WT mice, or between any age group of mice, in the amount of time each mouse spent in the open center zone of the open field behavioral task. Similarly, in the elevated plus maze task, both the APP/PS1 KI and WT mice spent more time in the enclosed arms compared with the open arms, and this was consistent across all age groups (Figure 2B). No significant differences were observed between APP/PS1 KI and WT mice at any age.

### Cognitive behavior

The cognitive behavior of APP/PS1 KI and WT mice was tested in the RAWM and NOR behavioral tasks - tests that measure spatial reference memory and recognition memory, respectively.

**Figure 3:**
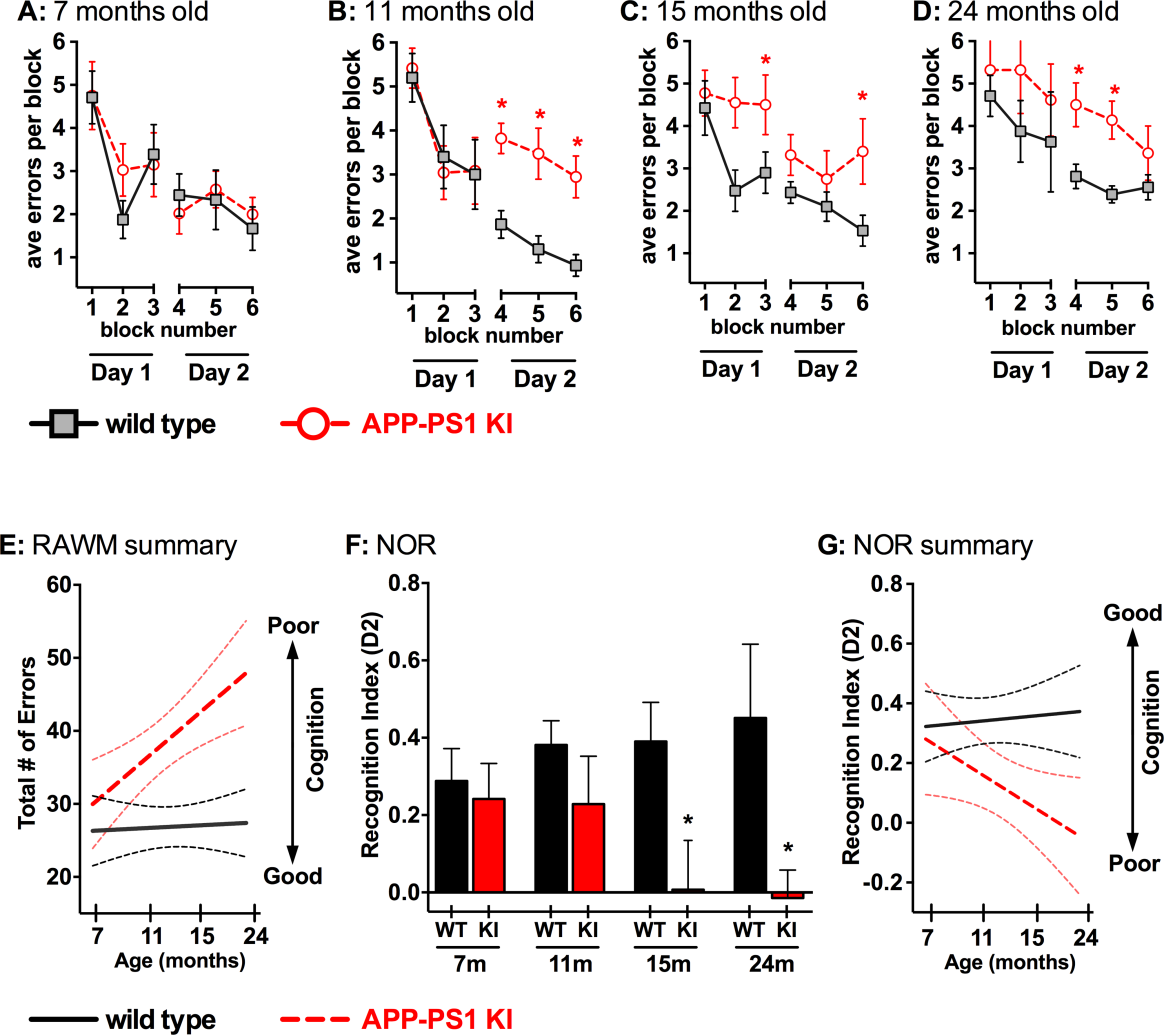
**Cognitive behavior of APP/PS1 KI and wild-type control mice**. Radial arm water maze (RAWM) performance for each age group: **(A) **no observable difference is seen between amyloid precursor protein/presenilin-1 knock-in (APP/PS1 KI) mice and wild-type (WT) mice at 7 months of age; however, cognitive deficits are observed in the APP/PS1 KI mice at **(B) **11 months, **(C) **15 months, and **(D) **24 months. **(E) **RAWM performance as a function of total errors across the four age groups. The WT cognitive performance remained relatively constant across the age groups, while APP/PS1 KI cognitive performance became more impaired as age increased. Data are plotted as mean (solid line) and confidence intervals (dashed lines). **(F) **Novel object recognition (NOR) performance in APP/PS1 KI and WT mice. The *D*^2 ^recognition index is used to control for total exploration time: *D*^2 ^= (novel - familiar)/(novel + familiar). Beginning at 15 months of age, the APP/PS1 KI mice show an inability to discriminate between objects while the WT mice are still able to discriminate. **(G) **NOR performance as a function of *D*^2 ^recognition index across the four age groups. The WT cognitive performance remained relatively constant across the age groups, while APP/PS1 KI cognitive performance became more impaired as age increased. *N *= 5 to 10 mice per group. *P <0.05, for APP/PS1 KI compared with WT. Number of mice: 10 WT and nine KI mice aged 7 months; five WT and 12 KI mice aged 11 months; 10 WT and 10 KI mice aged 15 months; and seven WT and five KI mice aged 24 months.

In the RAWM, there was no difference in performance between APP/PS1 KI mice and WT mice at 7 months of age (Figure 3A). In contrast, APP/PS1 KI mice aged 11 months old (Figure 3B), 15 months old (Figure 3C), and 24 months old (Figure 3D) performed significantly worse in the RAWM task than did the WT mice of the same ages. When the RAWM maze data are analyzed as a function of errors made versus age of the animals (Figure 3E), it is evident that APP/PS1 KI mice progressively increase the number of errors they make as they age (and the disease progresses).

In the NOR behavior task, the APP/PS1 KI and WT mice both showed a clear preference for novel object exploration during the dissimilar stimuli (A/B) session compared with the familiar at the ages of 7 and 11 months old (Figure 3F). However, in the groups of 15 and 24 month olds, the APP/PS1 KI mice lost the ability to discriminate between familiar and novel objects and were significantly impaired compared with the WT controls (*P *= 0.044 for 15 month olds, *P *= 0.041 for 24 month olds). Each animal tested had an exploration time >10 seconds with each object, and no significant difference in movement speed (velocity range: 2.4 to 3.6 cm/second) was observed between genotypes or age groups. When the NOR task data are analyzed as a function of the *D*^2 ^recognition index versus age of the animals (Figure 3G), it is evident that APP/PS1 KI mice also become progressively impaired in this task as they age and the AD-relevant pathology progresses.

## Discussion

Many lines of genetically altered mice have been generated during the past few decades in an effort to better understand the pathogenesis of AD. These mouse models of AD pathobiology have provided significant neuropathological, biochemical, physiological, and behavioral insights into AD pathogenesis. Many of the AD mouse models differ in the site of the mutation in APP, or in the number of APP mutations in one mouse, or the addition of mutations in PS1 or tau. In general, the genetic differences result in each mouse model having a unique pathobiology that makes it acutely useful for preclinical testing of a particular type of AD-relevant therapeutics. For example, if the mechanism of action of a specific preclinical therapeutic is to decrease cytokine overproduction, then a mouse model that demonstrates cytokine overproduction is needed for accurate testing of the therapeutic.

**Figure 4:**
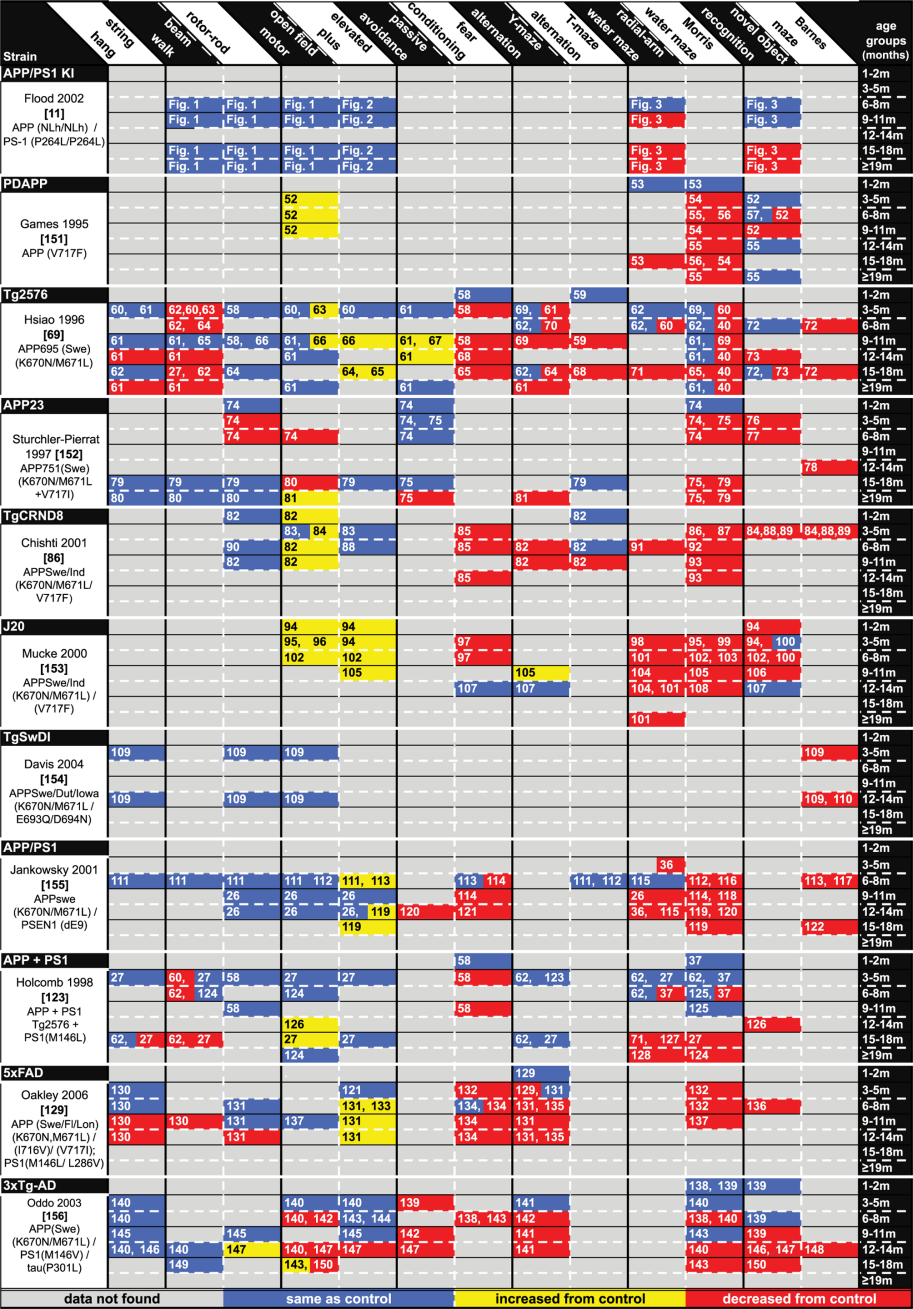
**Comparison of behavior changes in APP/PS1 KI mice with other common Alzheimer's disease mouse models**. Data analysis from the literature focused on a compilation of the most widely used behavioral tasks to assess the onset/progression of motor function impairment, anxiety-related behavior, and cognitive impairment. Blue data, the Alzheimer's disease (AD) mouse performed the same as the wild-type (WT) control; yellow data, the AD mouse was increased from control; red data, the AD mouse was decreased from control. The numbers within the data boxes refer to the literature citation. APP/PS1 KI, amyloid precursor protein/presenilin-1 knock-in.

Improvements in behavioral endpoints are often the gold standard used to demonstrate efficacy of preclinical therapeutics. However, for such endpoints to be useful, it is necessary to know when cognitive deficits reproducibly develop in the AD mouse model in relation to the particular druggable target of interest. As shown in Figure 4, the temporal time courses can be quite different between commonly used models. In addition, some behavioral tasks are clearly more sensitive to cognitive deficit detection at earlier ages. Figure 4 also shows how noncognitive behaviors, such as increased activity in motor tasks, should be considered in the selection of the most appropriate preclinical model to use for a particular study, because these noncognitive behaviors may interfere with the interpretation of cognitive tasks.

To assess the onset and progression of the cognitive impairments in the APP/PS1 KI mouse model, we have performed a comprehensive behavioral analysis across the lifespan of this model from 7 to 24 months old. Our data document clear age-dependent cognitive deficits in the APP/PS1 KI mice in both recognition memory (NOR) and spatial reference memory (RAWM) that become more prominent with increasing age. Further, in Figure 4 we compare the APP/PS1 KI mouse model with other AD mouse models in regard to the onset/progression of AD-relevant impairments in motor function, anxiety-related behavior, and cognition in an attempt to clarify how the APP/PS1 KI model fits into the growing literature on modeling AD in rodents. The data presented here for motor function, anxiety-related behavior, and cognitive deficits are critically important to the future application and utility of this model in preclinical testing of AD-relevant therapeutics by: establishing baseline behavioral characteristics of this model; and providing valuable information helpful in appropriate study design. These are important observations, as they establish baseline motor function and anxiety behavior, which is important to know in any mouse model prior to assessing cognitive functioning to avoid unwanted confounds [30,31].

In contrast to a number of the mice strains shown in Figure 4, which were found to have motor impairment and hyperactivity, the APP/PS1 KI mice exhibit no differences in ambulatory movement, grip strength, or coordination when compared with WT controls at any of the ages tested. There were also no anxiety-related behavior deficits seen at any age in the APP/PS1 KI mice in the elevated plus maze tasks. This is in contrast to the Tg2576, J20, APP/PS1, and 5xFAD mice, which all were found to have increased anxiety-related behavior in the elevated plus maze task. In the open field task, animals with elevated anxiety levels will spend little time exploring the center of the chamber [30,32]. The APP/PS1 KI mice do not exhibit anxiety-related behavior, as they showed normal exploration of the center of the open field chamber. Anxiety behavior did not differ between APP/PS1 KI mice and WT mice for any age tested. Our results therefore demonstrate that there are no deficits in either motor function or anxiety levels that could potentially confound cognitive testing in this mouse model of AD, which is dissimilar to the majority of the mouse models shown in Figure 4.

Attempts to model AD-relevant cognitive deficits in mouse models have met with some degree of success [33,34]. Reference memory is a type of memory that is often assessed in various mouse models of AD because deficits in this type of memory are highly specific for hippocampal function (one of the earliest/most severely affected brain regions in human AD) [35]. One common method for assessing deficits in hippocampal-based reference memory is through the use of exploration-based memory tasks such as the Morris water maze and the RAWM [33]. Here we observe deficits in spatial reference memory for the APP/PS1 KI mice when tested in the RAWM. These deficits in spatial reference memory are first evident in the 11 month age group, and continue to progress as the age/pathology increases in this model. The temporal onset and progression of cognitive impairment compared with other commonly used AD mouse models can be seen in Figure 4. For example, compared with other APP and PS1 AD mouse models, such as the APP/PS1 model and the APP + PS1 model, the onset of cognitive deficits in the APP/PS1 KI model appears to occur slightly later in life. Both the APP + PS1 model and the APP/PS1 model show deficits at 4 to 6 months of age [36-39], whereas the APP/PS1 KI mice show deficits starting at 11 months old (Figure 3).

Another commonly tested type of AD-relevant memory is that of recognition memory. The most common way to assess recognition memory in mice is through the use of the NOR task [33]. Here we see cognitive impairment in the NOR task manifest in the APP/PS1 KI model at 15 months of age. As shown in Figure 4, this temporal pattern is similar to the onset of NOR deficits in some other AD mouse models, such as the Tg2576 mice and the APP + PS1 model. In contrast, some mouse models such as the PDAPP, APP23, TgCRND8, J20, 5xFAD, and 3xTG-AD mice show impairment in recognition memory at a younger age. Overall, for many of the AD mouse models it appears that deficits in spatial working memory (Morris water maze and RAWM) become apparent earlier than deficits in recognition memory (NOR).

With regard to β-amyloid, similar to other commonly used models of AD such as the Tg2576 mouse and the 5xFAD mouse, in the APP/PS1 KI model the amyloid burden occurs prior to the onset of cognitive deficits [40-43]. Other potential therapeutic targets such as elevated levels of oxidative stress and neuroinflammation also occur before the observable cognitive deficits in the APP/PS1 KI mouse model [12,14,17,44]. With respect to oxidative stress, the APP/PS1 KI mouse model is also considerably different from other models such as the Tg2576 and 3xTG-AD mice in the magnitude and temporal time course of oxidative stress [15,44-48]. Elevated levels of oxidative stress have been linked to the cognitive deficits seen in AD [49,50]. In a study of the APP/PS1 KI mice [18], NADPH oxidase (a marker of oxidative stress) was reported to increase with the age of the animal and correlate with increased β-amyloid levels. Further, this same study reported that NADPH oxidase is correlated with APP/PS1 KI behavioral performance in the Stone T-maze, with lower levels of NADPH oxidase and slight deficits observable at 4 to 6 months of age and both becoming more pronounced by 16 to 19 months of age [18]. This provides an example of how the APP/PS1 KI mouse could be a useful model to test preclinical therapeutics targeted at reducing oxidative stress.

AD is a very complex disease, with many interrelated mechanisms and pathologies occurring concomitantly. Although no animal model fully replicates the human disease, AD mouse models are useful to investigate different aspects of AD pathology and disease progression. AD mouse models have been invaluable in advancing our understanding of disease mechanisms and in preclinical testing of potential therapeutics. Paramount to the selection of the appropriate model for evaluation of potential therapeutic interventions is demonstration that the desired relevant target for the preclinical therapeutic is present in the model at a therapeutically relevant time. For example, the APP/PS1 KI model used here is an ideal model for assessing the pathogenesis and effects of early oxidative stress or dysregulated neuroinflammation as we recently showed [17]. In contrast, the APP/PS1 KI model lacks AD-relevant tau pathology. This model would therefore be inappropriate to examine topics relating to the effect of microtubule-associated tau disruption in AD. Instead, a model such as the 3xTG-AD mouse (or another relevant model showing tau pathology) should be selected instead. Similarly, if AD-related motor deficits [51] are the target of research interest, selection of an appropriate model (Figure 4) showing motor deficits such as the Tg2576 or 5xFAD mouse would be salient, whereas a model like the APP/PS1 KI model that does not develop motor deficits would not be useful. Conversely, if AD-related cognitive deficits are of interest, then motor deficits need to be accounted for, or else a model that lacks motor deficits but that displays cognitive impairment should be selected so as to eliminate possible motor related confounds [31].

Each transgenic mouse model of AD provides different insights into aspects of AD pathology and progression. Careful forethought is therefore required in the selection of an optimal model based on specific research interests. Our hope is that, with regard to motor and cognitive deficits relating to AD, the discussion and Figure 4 presented here can help in the selection of the ideal model by providing an overview of the development and time course of behavioral deficits in the commonly used mouse models of AD.

## Conclusion

This study is the first comprehensive behavioral analysis reported for the APP/PS1 KI mouse model of AD and the results presented here add to a growing literature for this model. The lack of any motor/coordination deficits or abnormal anxiety levels, coupled with an age/disease-related cognitive decline and the high physiological relevance of this model, make it well suited for utilization in preclinical testing of AD-relevant therapeutics.

## Abbreviations

AD: Alzheimer's disease; APP: amyloid precursor protein; *D*^2^: discrimination index; KI: knock-in; NADPH: nicotinamide adenine dinucleotide phosphate; NOR: novel object recognition; PS-1: presenilin-1; RAWM: radial arm water maze; WT: wild type

## Competing interests

The authors declare that they have no competing interests.

## Authors' contributions

SJW participated in the design of the research studies, performed the behavioral tests, and drafted the manuscript. ADB participated in the design of the study, performed the statistical analyses, and helped analyze data and draft the manuscript. LJVE conceived of the study, participated in its design and coordination, and helped write the manuscript. All authors read and approved the final manuscript.
